# The Impact of Yoga Practice on Health, Strength, and Respiratory Capacity in Portuguese Airforce Pilots: an Applied Psychophysiology and Biofeedback Approach

**DOI:** 10.1007/s10484-025-09719-9

**Published:** 2025-06-13

**Authors:** Sara Santos, Santos Villafaina, José Alberto Parraca, Orlando Fernandes, Filipe Melo

**Affiliations:** 1 Departamento de Desporto e Saúde, Escola de Saúde e Desenvolvimento Humano, Universidade de Évora, 7004-516 Évora, Portugal; 2 Comprehensive Health Research Centre (CHRC), University of Évora, 7004-516 Évora, Portugal; 3 School of Health Sciences, University of Algarve (ESSUAlg), Campus da Penha, 8005-139 Faro, Portugal; 4 Facultad de Ciencias del Deporte, Universidad de Extremadura, 10003 Cáceres, Spain; 5 Laboratório de Comportamento Motor, Faculdade de Motricidade Humana, Universidade de Lisboa, 1649-004 Lisbon, Portugal

**Keywords:** Physical conditioning, Physical performance, Hand grip strength, Total lung capacity, Forced expiratory volume, Work performance, Military health

## Abstract

Top performance in military aviation relies on strong health. Handgrip strength is key, showing overall strength and work capacity. Since rarefied air affects focus and mission success, respiratory training is essential. The impact of Ashtanga Vinyasa Yoga on Portuguese Air Force Academy pilots from the 2021 and 2022 classes was assessed, aiming to enhance health, lung capacity, and strength. A randomized controlled trial involved 18 individuals from the "Masters in Military Aeronautics: aviator pilot specialist". Participants were randomly assigned to yoga classes (intervention n=10) or a waiting list (control n=8). General health, strength, and respiratory capacity were measured using SF-36 questionnaire, a hand-grip dynamometer, and a spirometer, respectively, before and after a 12-week yoga program. Parametric and non-parametric tests were conducted using Jamovi (version 2.3.26). ClinicalTrials.gov identifier NCT05821270, registered on April 19, 2023.Revealed significant within-group differences pre- vs post-intervention for general health, handgrip strength, and FEV1%. There was a significant difference between groups for lung capacity.Yoga participants showcased superior results versus controls, suggesting yoga's positive impact. The yoga protocol, in conjunction with military training, positively affected health, strength, and lung performance, highlighting its operational benefits even in highly trained individuals.

## Introduction

Yoga, an ancient practice promoting health and well-being, is based on four key principles (Desikachar et al., 2005):

1. Views the human body holistically, considering it a unified system.
2. Acknowledges that everyone is unique, so personalized approaches are important.
3. Encourages self-empowerment, with practitioners playing an active role in their own journey.
4. Highlights the importance of one’s mental state in the healing process.

Yoga doesn't advocate a one-size-fits-all approach. The dosage of asana (physical postures) and pranayama (breathing techniques) for healing varies based on the constitution of the individual (Desikachar et al., 2005). Incorporating both ásana and pranayama practices yields more notable improvements compared to isolated pranayama exercises (Balaguru et al., 2021; Dubey et al., 2022). Combining asanas with pranayama offers enhanced benefits in lung capacity, muscle strength, respiratory endurance, and mental well-being compared to isolated pranayama exercises:

- Combined asana and pranayama practices can lead to greater increases in vital capacity, which reflects the lungs’ ability to hold more air, thus improving overall respiratory efficiency (Balaguru et al., 2021).
- The combination enhances both inspiratory and expiratory pressures more effectively than pranayama alone, contributing to stronger respiratory muscles (Balaguru et al., 2021).
- Integrating asanas with pranayama significantly boosts respiratory endurance, which helps maintain efficient breathing over prolonged periods, an essential factor for athletes and healthy individuals alike (Balaguru et al., 2021).
- Ásanas complement pranayama by stretching and expanding the chest, diaphragm, and other muscles involved in breathing, leading to improved lung capacity and efficiency (Dubey et al., 2022).
- In addition to physical benefits, the combined practices promote better stress management, mental clarity, and emotional stability due to their calming effect on the nervous system, which is more profound than with pranayama alone (Dubey et al., 2022).

This integrated approach requires careful and intelligent adaptation to address specific issues or objectives that the practitioner wants to tackle or where they need support (Balaguru et al., 2021; Dubey et al., 2022). The array of healing tools available in yoga, such as combinations of techniques designed by qualified instructors offers numerous possibilities. These tools must be thoughtfully combined to suit the unique needs of each practitioner (Desikachar et al., 2005; Dubey et al., 2022). Given the professional needs of the pilot students of the Portuguese Air Force Academy, this study proposes to tailor a specific practice to this population and determine if the practice of a comprehensive yoga program will improve physical functioning in pilots, which are important to successfully conducting flight missions (Choudhary, 2023; Dubey et al., 2022; Mandanmohan et al., 2003; Santos et al., 2024; Thangavel et al., 2014). Hand-grip force has been used as an objective clinical measure in a variety of situations including the perception of the overall strength to determine an individual's ability to work. It is influenced by effort, contractility, and skeletal muscle mass (Dubey et al., 2022; Mandanmohan et al., 2003; Santos et al., 2024). As a physiological, objective, and accurate muscle test, the hand-held dynamometer is an indicator of muscle function and nutritional status (Dubey et al., 2022; Mandanmohan et al., 2003; Santos et al., 2024). Spirometry is a gold standard lung function test that asserts how an individual inhales or exhales air volumes through a period of time (Durmic et al., 2015; American Thoracic Society, 1991). This test assesses pulmonary capacities by measuring various variables, based on standardized respiratory maneuvers, including:

1. Forced vital capacity (FVC),
2. Forced expiratory volume in one second (FEV_1_),
3. Lung volumes,
4. Flow rates.

It provides a comprehensive assessment of pulmonary performance by comparing these metrics with reference standards for height, sex, and age. This allows for determining the existence of airflow obstruction or whether lung volumes are normal. Such exercises have a significant impact on the physiological adaptation of the respiratory system (Durmic et al., 2015; American Thoracic Society, 1991).

Spirometry (a test that measures lung function by assessing the volume and flow of air during inhalation and exhalation) and respiratory training methods take on heightened significance in the context of military pilots, as there is a scarcity of studies focusing on specialized spirometric measures tailored to their needs (Bustamante-Sánchez et al., 2021; MacIntyre et al., 1981; Whitley, 1997). This is especially pertinent when considering scenarios where pilots operate aircraft at altitudes characterized by rarefied air, necessitating the use of oxygen bottles to sustain flight autonomy. Then, it becomes crucial not only for maintaining operational times during flight but also for sustaining focus and optimal mission performance in environments with diminished oxygen levels. Regarding respiratory training, while a military aviation career does not significantly correlate with lung volume increases, the frequency of supranormal pulmonary function test results among military personnel has decreased over time (Bustamante-Sánchez et al., 2021; MacIntyre et al., 1981; Whitley, 1997). Therefore, the interpretation of pulmonary function tests in active-duty personnel undergoing assessment should align with that of typical patients (Cochet et al., 2014). Additionally, the potential respiratory risks associated with exposure to military weapon smoke during live shooting training or enclosed combat environments must be acknowledged (Borander et al., 2017).

Considering these factors, there might be notable advantages in integrating pranayama-based respiratory training into the routine of this population, given that it is not typically included in regular military training (Borander et al., 2017; Severo et al., 2006). This practice can potentially address unique challenges posed by the military context and contribute to enhanced respiratory function and overall well-being.

The SF-36 V1 questionnaire (Severo et al., 2006; White et al., 2018) will indicate if the exercise protocol had any impact in the pilot’s general health subjective assessment, mitigating adverse effects of their profession on pilots’ physical well-being.

The Portuguese Air Force’s Health and Physical Exercise Department is currently in the stages of formulating a compulsory sports training regimen for military pilots. To our knowledge, pilots have thus far been advised to engage in physical training according to their discretion, with the goal of meeting the required Portuguese Airforce standards for mandatory annual physical assessments. The present study delved into the impact of ashtanga vinyasa yoga on hand-grip force dynamometry and spirometry among adult, healthy student pilots enrolled at the Portuguese Air Force Academy. The underlying premise was that yoga could potentially result in enhancements in lung capacity and overall strength, subsequently warranting its inclusion in a mandatory sports program initiative. Incorporating yoga into a structured, mandatory training program could better prepare pilots for these mandatory physical assessments, offering a holistic approach to meet the physical and mental demands of military aviation. The goal is to address key challenges in pilot training—enhancing lung capacity, strength, cardiovascular fitness, and stress management, all critical for passing the mandatory annual physical evaluations as well as to increase flight safety.

## Materials and Methods

The methodology used in this randomized controlled trial builds upon the detailed procedures previously described in our published methods article (Santos et al., 2024).

Human participants review was made by the Évora University research ethics committee, with approval number 21050. The study was registered at ClinicalTrials.gov and received the identifier NCT05821270, on 19 th April 2023. Informed consent was obtained from all pilots involved in the study.

The study aimed to evaluate strength and respiratory capacity changes before and after the three-month period. The participants were randomly assigned, first giving each pilot a number and then using random.org, to insert the pilots either in the “yoga class” intervention group (*n* = 10) or the “waiting list” control group (*n* = 9). Besides standard military training, mandatory for all pilots, the intervention group engaged in a 12 week program of ashtanga vinyasa yoga supta, while the control group pursued only standard pilot training, involving theoretical and practical flight exercises, mission planning and execution, and debriefing.

The yoga group classes followed a structured format encompassing five key elements (Desikachar et al., 2005; Santos et al., 2024), all adapted to pilot’s personal capacity:

1. A five-minute segment of *prathyáhara* (focused attention),
2. A five-minute segment of *pranayama* (comprising the classic *raja yoga pranayama* techinque–that involves the four phases of the breath, Inhale or *Pūraka*; Internal breath retention or *Antar Kumbhaka*; Exhale or *Rechaka* and External breath retention or Bahya Kumbhaka.– and *kriya* breathwork techniques– *Nauli Kriya* in which one exhales fully, holding the breath out, and practices abdominal contractions leading to isolating and rotating the abdominal muscles in circular motion with focus on breath control, detoxification, and strengthening the core),
3. A 35-minute segment of *asana* (physical postures involving various spinal movements and inversions),
4. An additional five-minute period of *dháraná* (initially incorporating *yoga nidra* relaxation and then transitioning to mental exercises to enhance focus for meditation),
5. And a concluding five-minute period of *dhyána* (meditation).

These five components were selected and adjusted in order and duration to suit the specific requirements of the pilot group during their flight missions (Desikachar et al., 2005; Santos et al., 2024). This sequencing, supported by classical yoga literature, facilitates mental centering and autonomic balance, enhancing the effects of subsequent physical postures (Saraswati, 1996; Swami Kuvalayananda, 2015). All yoguic techniques progressed in complexity over the 12-week program. Breathing cycles were initially guided but allowed for self-regulation, promoting adaptability to individual physiological states—a core principle in traditional pranayama theory and modern integrative health research (Jerath et al., 2006; Telles et al., 2013). This approach also aligns with evidence suggesting pranayama improves cardiorespiratory control and reduces sympathetic activity, contributing to enhanced postural and psychological resilience (Sivaramakrishnan et al., 2021).

Within this framework, two distinctive aspects were incorporated: *supta* and *ashtanga vinyasa*. The *supta* approach involved conducting the class predominantly or entirely with closed eyes, a technique designed to minimize visual system responses while eliciting vestibular system responses. The *ashtanga vinyasa* component featured fluid movement sequences, emphasizing synchronized breathwork and primarily targeting the enhancement of neck and upper limb strength. This multifaceted approach was implemented with the intention of optimizing the benefits for the pilot group, adapting the yoga practice to their specific flight mission needs (Balaguru et al., 2021; Dubey et al., 2022; Mandanmohan et al., 2003; Thangavel et al., 2014).

The intensity of classes was evaluated through the Borg scale in which each participant evaluated their effort (Borg, 1990). This allows participants to subjectively assess their physical effort based on how they feel. We asked participants to rate their perceived level of exertion during the class on a scale from 6 to 20, where 6 indicates “no exertion at all” and 20 represents “maximum exertion.”

Pilots reported their body height in accordance to their official identification documents (*Cartão do Cidadão*) and weight was measured using an electric bioimpedance scale (Tanita, MC-780 MA, Tanita, Tokyo, Japan).

The handgrip force was evaluated using a handgrip dynamometer (Baseline Smedley, Model 12–0286, White Plains, NY, USA). The test was conducted in a bipodal position, and the dominant arm tested next to the body with flexion of about 90º of the elbow. The dynamometer was adjusted to the width of each individual’s hand. Participants were instructed to grip the dynamometer with maximum isometric effort for 3 s, with 3 attempts to the dominant hand and about 30 s apart. The mean of the attempts was used to determine the handgrip strength of each individual (Santos et al., 2024).

Respiratory capacity was analyzed with a spirometer (Vitalograph copd-6™) before and after a 12-week yoga program. The spirometry reference values are derived from a population similar to the study participants (young Caucasian adults), with the same type of assessment instrument and protocol used in this reference document (Pellegrino, 2005; David and Edwards, 2022). *Forced Vital Capacity* (FVC) was evaluated with a spirometer (Vitalograph copd-6™), and each individual was asked for a long inspiration, followed by strong and long expiration through the mouthpiece of the device and these values were recorded. Another volume evaluated and extremely important is the FEV1, which corresponds to the amount of air eliminated in the first second of the forced expiratory maneuver, as well as the FEV6, which corresponds to the amount of air eliminated in the first six seconds of the forced expiratory maneuver (Pellegrino, 2005). FEV1 and FEV6 are critical respiratory measures for military aviation pilots because they assess lung function and the efficiency of air expulsion, which directly impact pilots’ ability to withstand the physical demands of high-altitude, high-stress environments (Bustamante-Sánchez et al., 2021; Cochet et al., 2014; Durmic et al., 2015; MacIntyre et al., 1981). FEV1 reflects the volume of air a person can forcibly exhale in the first second of a breath. For military pilots, this is crucial as it indicates the capacity to rapidly expel air under conditions of high-pressure changes, such as during rapid altitude adjustments or anti-G force maneuvers. FEV1 examines if there’s robust lung function and quick airway responsiveness, which can help mitigate hypoxia and other altitude-related respiratory issues (Bustamante-Sánchez et al., 2021; Cochet et al., 2014; Durmic et al., 2015; MacIntyre et al., 1981). FEV6 measures the total volume of air expelled in six seconds and serves as a proxy for Forced Vital Capacity (FVC). For pilots, FEV6 is important as it evaluates the ability to maintain prolonged exhalation, which can be necessary during sustained high-pressure conditions, such as during long combat flights. FEV6 examines sustained lung function and endurance, crucial for preventing fatigue and maintaining alertness (Bustamante-Sánchez et al., 2021; Cochet et al., 2014; Durmic et al., 2015; MacIntyre et al., 1981). With yoga’s emphasis on breath control and mindfulness (Desikachar et al., 2005), we expect improvement in these critical lung function parameters, in order to help pilots perform better under demanding flight conditions, maintain endurance, and reduce respiratory strain.

The pilot’s general health was assessed with the SF-36 V1 questionnaire, administered before and after the 12-week yoga program. This self-report instrument measures health-related quality of life across various domains, including physical functioning, role limitations due to physical health, bodily pain, general health perceptions, vitality, social functioning, role limitations due to emotional problems, and mental health. The SF-36 questionnaire scores each domain on a scale ranging from 0 to 100, with higher scores indicating better health-related quality of life (Severo et al., 2006; White et al., 2018; Ware, 1993).

In this study, pilots were shown their biofeedback results on handgrip strength and spirometry, tested immediately after a day of military work and right before a flight emergency simulation, before and after the 12 weeks, to help them use self-regulation techniques to improve their health and performance.

Data was analyzed with Jamovi (version 2.3.26), using parametric and non-parametric tests: differences at baseline were studied using the t-test for paired samples (Kim, 2015) with p values of less than 0.05 accepted as significant and Wilcoxon signed rank test (Wilcoxon, 1945) with p values of less than 0.05 accepted as significant, according to Shapiro-Wilk normality test results: normal data distribution was accepted with p values greater than 0.05 (Rosner et al., 2006). Additionally, the effect sizes were calculated for each comparison. Cohen’s term *d* classified effect sizes as small (*d* = 0.2), medium (*d* = 0.5), and large (*d* ≥ 0.8). According to Cohen, “a medium effect of 5 is visible to the naked eye of a careful observer” (Fritz et al., 2012; Ho et al., 2019; Sullivan & Feinn, 2012).

The *Tirocinium* is the final stage of military aviation training and takes place in Base Aérea Nº11, in Beja, Esquadra 101. It lasts 10 months, with 18 days of theoretical instruction and 185 days of practical instruction (177 h in aircraft and 33 h in a stationary flight simulator, for a total of 180 flight hours per pilot).

**Fig. 1:**
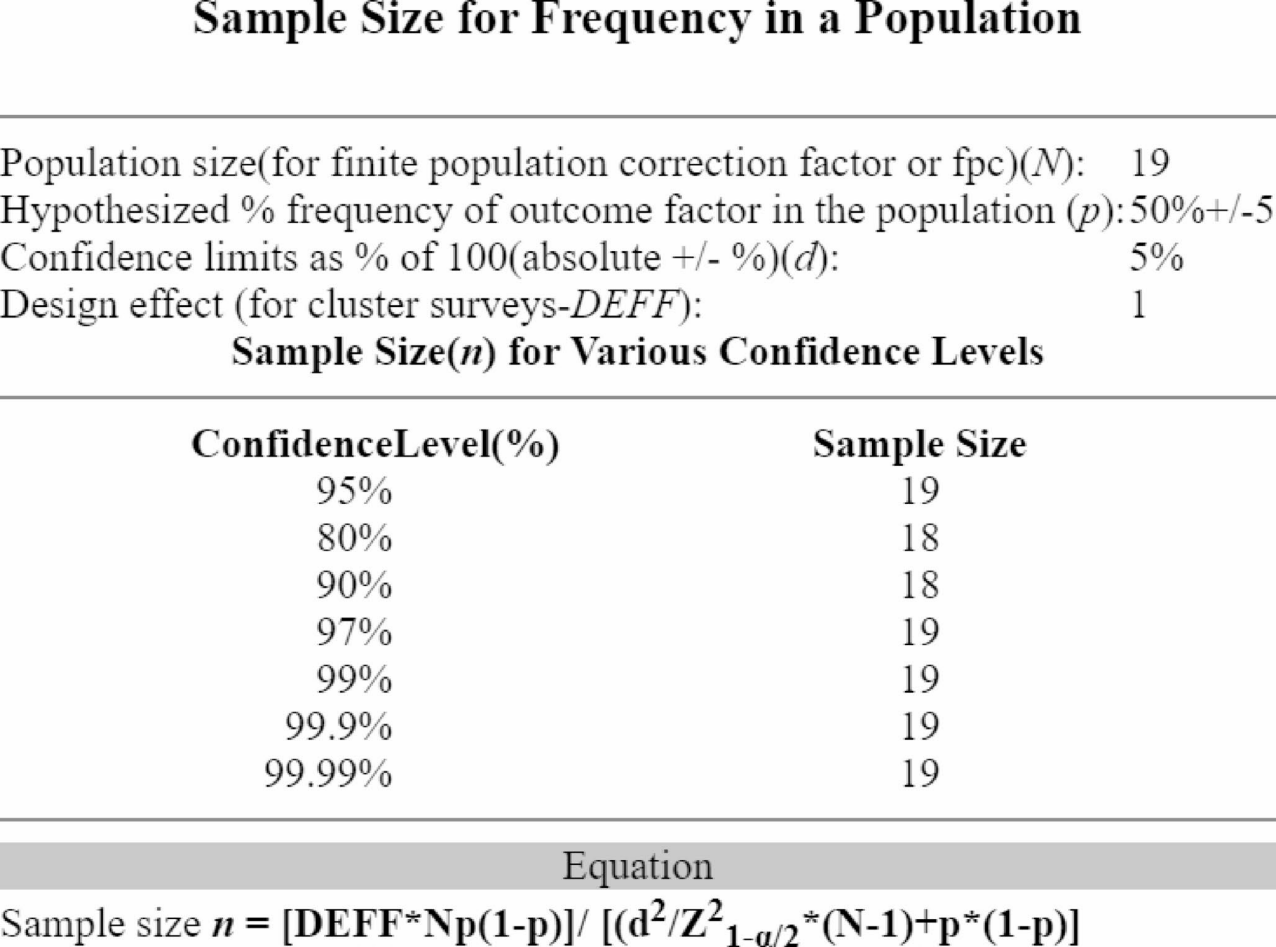
Sample Size results from OpenEpi, Version 3, open source calculator—SSPropor

All pilots in the early stages of their *Master in Military Aeronautics: Aviator Pilot Specialization* course (classes of 2021 and 2022) at the Portuguese Air Force Academy were involved in this research. Sample size calculation is shown in Fig. 1.

The frequency and duration of yoga practice were customized according to the pilots’ work schedule, involving two sessions per week, each lasting one hour. These sessions were tailored to address perceived challenges faced by pilots.

The following perceived challenges faced by pilots were obtained through an interview with the F-16 team captain: hypoxia remains the most important hazard on high-altitude flights as well as increased G-forces due to improvements in aircraft ergonomics. These factors increase the height and speed of flights, enhancing the need of a better adaptations of the psychophysiological response (Whitley, 1997; Bustamante-Sánchez et al., 2021).

Uniformity was maintained across participants in terms of provided meals by the Portuguese Air Force and equivalent working hours. The pilots were instructed to adhere to their established fitness routines (as per the requirements for passing professional physical tests) and their daily habits (including diet, caffeine or medication intake, sleep schedule, etc.) throughout the study duration. One pilot was excluded from the control group (*n* = 8) due to significant lifestyle changes during the three-month timeframe, including alterations in diet, exercise, and smoking habits.

## Results

### General Composition

Descriptive data for pilot’s general body composition at baseline and after the 12-week program are presented in Table 1 with Mean (Standard Deviation) values.

**Table 1:** Descriptive statistics of the pilots’ body composition in both yoga and control groups at baseline and after 12 weeks

		Pre-intervention Mean (SD)	Post-intervention Mean (SD)
Age (years)	Control group	23.9 (0.78)	24.3 (0.87)
	Yoga group	24.6 (1.17)	24.9 (1.37)
Height (cms)	Control group	178 (4.27)	178 (4.27)
	Yoga group	176 (4.60)	176 (4.60)
Weight (kgs)	Control group	80.8 (5.66)	82.3 (6.12)
	Yoga group	74.1 (8.84)	74.7 (9.74)
IMC	Control group	24.5 (2.41)	25.9 (1.76)
	Yoga group	23.8 (2.49)	24.0 (2.8)
Fat mass (kgs)	Control group	14.7 (2.19)	15.4 (2.57)
	Yoga group	13.3 (3.04)	12.9 (4.18)

### Borg CR10 Scale

**Fig. 2:**
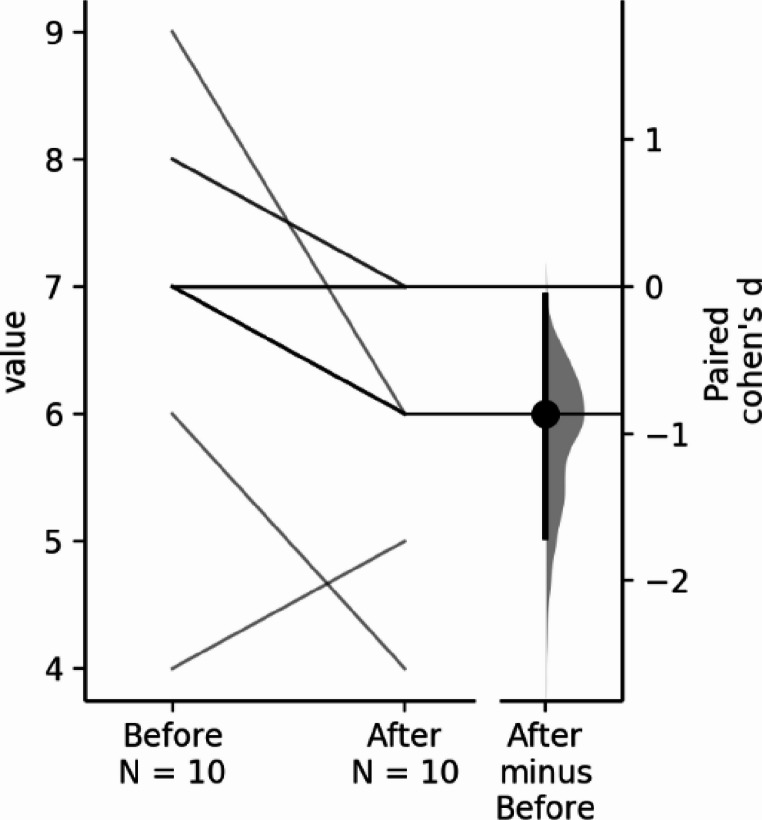
Borg effect size: the paired Cohen’s d between Borg Before and Borg After is −0.866 (95.0%CI −1.7, −0.0657), with the P value of the two-sided permutation t-test being 0.0034 (Ho et al., 2019)

Borg CR1O scale descriptive statistic values Mean (SD) pre-intervention were 7.00 (1.33) and post-intervention changed to 6.00 (0.94). Data had a normal distribution with Shapiro-Wilk W being 0.872 and *p* = 0.105 so parametric statistical tests were used, the paired samples t-test results are 3.00 with a *p* = 0.015, showing a significant difference in the intervention group, comparing baseline values with the post 12-week yoga intervention values.(Fig 2)

### Strength

Descriptive data for pilot’s handgrip strength at baseline and after the 12-week program are presented in Table 2. Shapiro-Wilk normality tests were conducted showing the data has a normal distribution for the control group but not for the intervention group. Since the handgrip data set did not have a normal distribution, non-parametric statistical tests were used, the Wilcoxon signed rank test results show a significant difference in the intervention group but not in the control group for the handgrip strength comparing baseline values with the post 12-week yoga intervention values. To compare possible differences between control group and yoga group before and after an intervention Wilcoxon signed rank test was also used with no significant results found.

**Table 2:** Results for the handgrip strength values

Descriptive statistics			
		Pre-intervention Mean (SD)	Post-intervention Mean (SD)
Control group		45.0 (3.82)	46.5 (4.48)
Yoga group		41.2 (8.70)	44.9 (8.06)
**Shapiro-Wilk normality tests**			
		W	p
Control group Pre vs. post intervention		0.924	0.466
Yoga group Pre vs. post intervention		0.844	0.049
**Wilcoxon W within groups**			
		W	p
Control group		8.00	0.181
Yoga group		0.00	0.006
**Wilcoxon W between groups**			
		W	p
Control group vs. Yoga group Pre intervention		22.0	0.641
Control group vs. Yoga group Post intervention		16.0	0.844

**Fig. 3:**
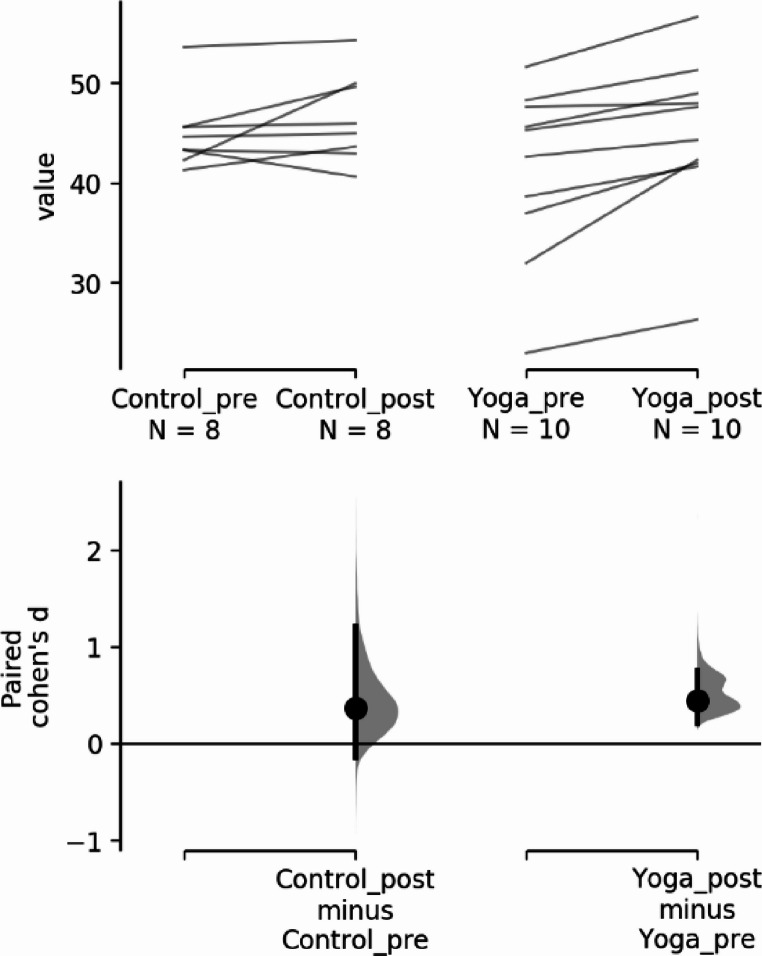
Handgrip strength for the paired Cohen’s d for 2 comparisons are shown in the above Cumming estimation plot (Ho et al., 2019)

In terms of effect size, (Fig. 3), for handgrip strength:

- The paired Cohen’s d between Control_pre and Control_post is 0.371 (95.0%CI −0.144, 1.22) and the P value of the two-sided permutation t-test is 0.164.
- The paired Cohen’s d between Yoga_pre and Yoga_post is 0.445 (95.0%CI 0.213, 0.757) and the P value of the two-sided permutation t-test is 0.0018.

### Spirometry

Descriptive statistics on Lung Capacity, FEV1% and FEV6% are shown in Table 3. Shapiro-Wilk normality tests were conducted showing the data has a normal distribution for FEV1% but not for Lung Capacity or FEV6%. FEV1% data had a normal distribution so parametric statistical tests were used, the t-test for paired sample’s results show a significant difference in the intervention group but not in the control group comparing baseline values with the post 12-week yoga intervention values. For the Lung Capacity and FEV6% data the Wilcoxon signed rank test was used and showed significant results in FEV6% within both control and yoga group. The same test was used to compare groups before and after intervention, revealing significant results in lung capacity and FEV6% post intervention, as well as FEV1% pre intervention.

**Table 3:** Results for lung capacity, FEV1% and FEV6% within and between groups

Descriptive statistics						
					Pre-intervention Mean (SD)	Post-intervention Mean (SD)
Lung capacity				Control group	20.25 (0.71)	20.00 (0.00)
				Yoga group	26.30 (6.82)	33.00 (13.48)
FEV_1_%				Control group	107.13 (3.60)	120.75 (24.55)
				Yoga group	95.30 (12.07)	119.00 (25.80)
FEV_6_%				Control group	99.50 (5.07)	123.00 (23.00)
				Yoga group	107.30 (20.90)	90.30 (9.32)
**Shapiro-Wilk normality tests**						
					W	p
Lung capacity				Control group Pre vs. post intervention	0.418	< 0.001
				Yoga group Pre vs. post intervention	0.770	0.006
FEV_1_%				Control group Pre vs. post intervention	0.980	0.961
				Yoga group Pre vs. post intervention	0.854	0.064
FEV_6_%				Control group Pre vs. post intervention	0.882	0.198
				Yoga group Pre vs. post intervention	0.818	0.024
**Student’s T tests for paired samples within groups**						
				statistic	df	p
FEV_1_%	Control group Pre vs. post intervention			−1.69	7.00	0.135
	Yoga group Pre vs. post intervention			−2.91	9.00	0.017
**Wilcoxon W within groups**						
					W	p
Lung capacity		Control group Pre vs. post intervention			1^a^	1.000
		Yoga group Pre vs. post intervention			8.00^b^	0.183
FEV_6_%		Control group Pre vs. post intervention			1	0.021
		Yoga group Pre vs. post intervention			42.00^c^	0.024
**Wilcoxon W between groups**						
					W	p
Lung Capacity			Control group vs. Yoga group Pre intervention		0.00^d^	0.181
			Control group vs. Yoga group Post intervention		0.00^e^	0.058
FEV_1_%			Control group vs. Yoga group Pre intervention		33.00	0.042
			Control group vs. Yoga group Post intervention		−13.00	0.528
FEV_6_%			Control group vs. Yoga group Pre intervention		6.50	0.123
			Control group vs. Yoga group Post intervention		33.00	0.039

^a^seven pairs of values were tied; ^b^two pairs of values were tied; ^c^one pair of values were tied; ^d^five pairs of values were tied; ^e^three pairs of values were tied.

**Fig. 4:**
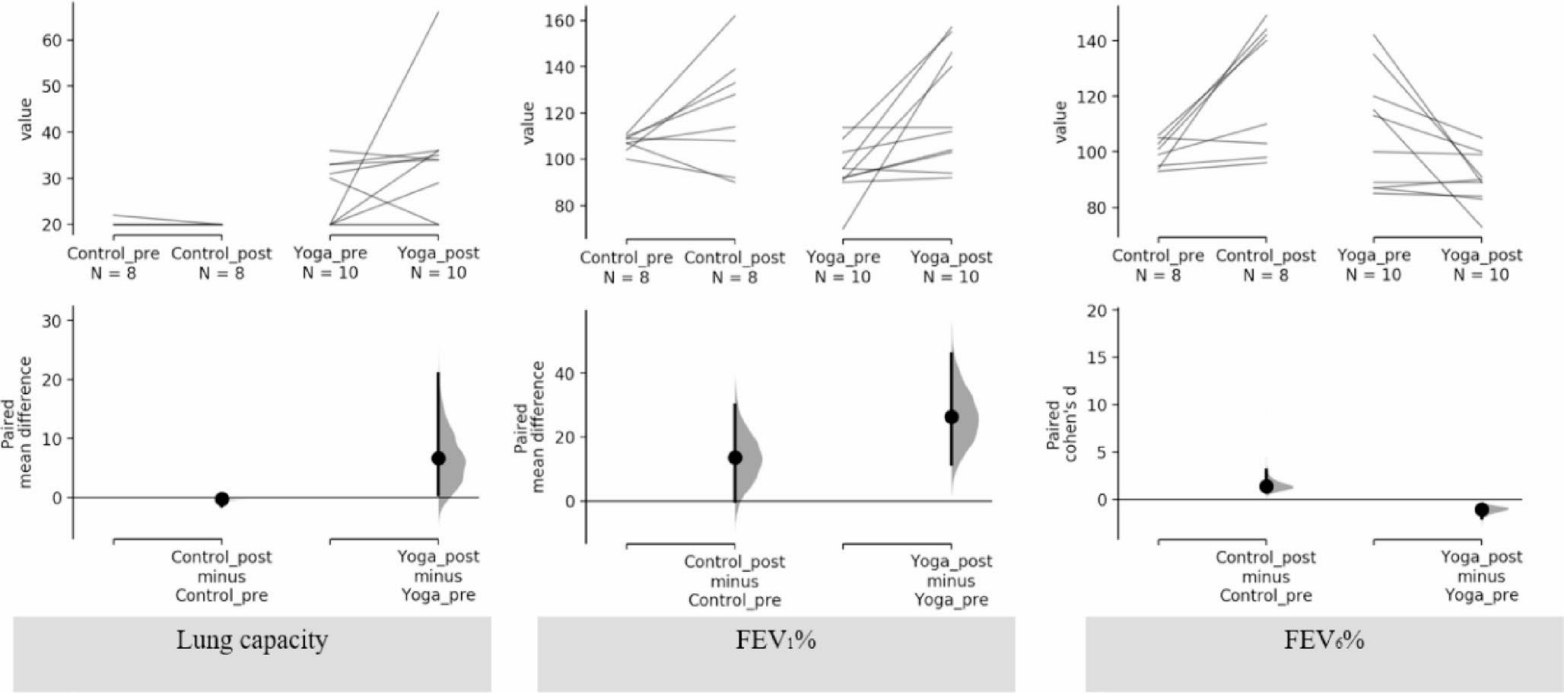
Handgrip Lung capacity, FEV1% and FEV6% paired mean difference comparisons are shown in the above Cumming estimation plots (Ho et al., 2019)

For Lung Capacity’s effect size (Fig. 4): the paired mean difference between Control_pre and Control_post is −0.25 (95.0%CI −1.5, 0.0) and the P value of the two-sided permutation t-test is 0.0; the paired mean difference between Yoga_pre and Yoga_post is 6.7 (95.0%CI 0.5, 21.0) and the P value of the two-sided permutation t-test is 0.16. In terms of effect size, for FEV1%: the paired mean difference between Control group pre and post 12 weeks is 13.6 (95.0%CI 0.0, 30.0) and the P value of the two-sided permutation t-test is 0.127; the paired mean difference between Yoga group pre and post 12 weeks is 26.4 (95.0%CI 11.4, 46.0). The P value of the two-sided permutation t-test is 0.0104. In relation to FEV6% effect size: the paired Cohen’s d between Control_pre and Control_post is 1.4 (95.0%CI 0.735, 3.08) and the P value of the two-sided permutation t-test is 0.008. The paired Cohen’s d be-tween Yoga_pre and Yoga_post is −1.05 (95.0%CI −1.99, −0.481) and the P value of the two-sided permutation t-test is 0.0106 (Table 4)

**Table 4:** Results of the SF-63 questionnaire

Descriptive statistics			
		Pre-intervention Mean (SD)	Post-intervention Mean (SD)
Control group		86.7 (7.56)	78.8 (12.09)
Yoga group		81.8 (11.27)	84.3 (9.49)
**Shapiro-Wilk normality tests**			
		W	p
Control group Pre vs. post intervention		0.881	0.194
Yoga group Pre vs. post intervention		0.884	0.144
**T-test within groups**			
		statistic	p
Control group		2.09	0.75
Yoga group		−1.57	0.150
**T-test between groups**			
		statistic	p
Control group vs. Yoga group Pre intervention		0.889	0.404
Control group vs. Yoga group Post intervention		−0.995	0.353

### General Health Subjective Assessment (SF-36 V1 questionnaire)

**Fig. 5:**
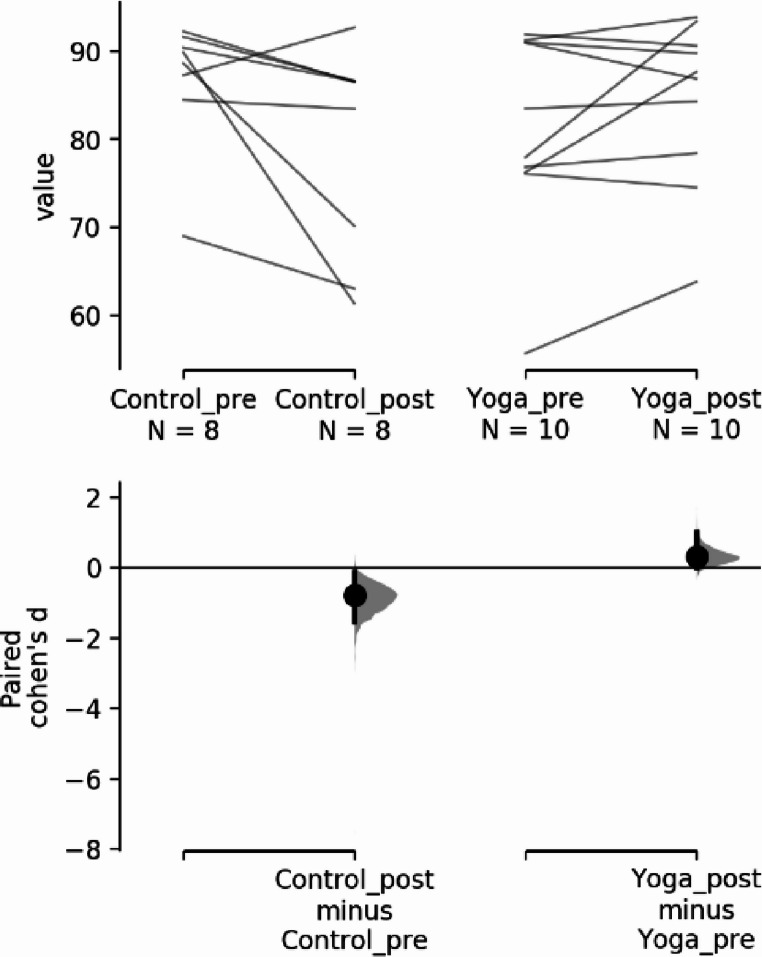
SF-36 questionaire total results paired mean difference comparisons are shown in the above Cumming estimation plots (Ho et al., 2019)

The paired Cohen’s d between control group before intervention and control group after intervention is −0.782 (95.0%CI −1.53, −0.134), with a P value of the two-sided permutation t-test of 0.0394. The paired Cohen’s d between intervention group before yoga and intervention group after yoga is 0.304 (95.0%CI −0.00642, 1.0), with a P value of the two-sided permutation t-test of 0.18. (Fig. 5).

## Discussion

Results revealed significant positive within-group differences pre- vs. post-intervention for general health, handgrip strength, and FEV1% for the yoga group and a significant difference between groups for lung capacity showcasing yoga as beneficial to pilot’s military training.

### Strength

The intervention group’s p value signifies systematic effect due to yoga despite control group’s higher baseline strength, linked to weight and height. Yoga combines stretching, balance, and isometric exercises engaging various muscles, including hands and arms (Dubey et al., 2022; Mandanmohan et al., 2003; Thangavel et al., 2014). Controlled breathing and mindfulness might enhance muscle engagement, neuromuscular coordination, and grip performance, supported by prior research (Dubey et al., 2022; Mandanmohan et al., 2003; Thangavel et al., 2014). Control group’s lack of change could relate to absent structured exercise intervention, baseline variation, and uncontrolled factors (Miranda et al., 2022). Yoga group’s Cohen’s d signifies stronger effect size, aligning with Wilcoxon test’s significance, highlighting substantial yoga impact. Conversely, the control group didn’t show significant handgrip strength differences, while the intervention group exhibited notable improvement, suggesting yoga’s positive impact. The control group’s non-significant p value indicates potential chance or variability in handgrip strength changes.

### Spirometry and Perceived Effort

This study found no significant lung capacity differences within groups for either the intervention or control groups when comparing baseline to post 12-week values. The effect size for lung capacity reveals a large change in the intervention group, but a small change in the control group. Lack of significance could relate to prior pilot training (Cochet et al., 2014; MacIntyre et al., 1981).

Comparing between groups, no pre-intervention differences were found, yet post-intervention values showed significance favoring the intervention group and showcasing that combining yoga with military training significantly affected lung performance. The value suggests post-intervention yoga group’s higher lung capacity. Essentially, yoga-practicing participants increased lung capacity, aligning with results from larger studies with varied pranayama techniques (Whitley, 1997; Abel et al., 2013; Dinesh et al., 2015; Dubey et al., 2022; Mandanmohan et al., 2003; Sheetal et al., 2012; Thangavel et al., 2014).

Significant FEV1% difference emerged post 12-week yoga intervention in the intervention group, but not in the control group when comparing baseline and post-intervention values. For effect size, intervention and control groups both displayed large effect sizes for pre- to post-intervention mean differences. Despite insignificance, large effect size implies regular military training impacted the control group’s lung function. In the intervention group, FEV1% significantly improved, with higher post-intervention SD. Yoga enhanced lung function in intervention group, yielding varying responses: some pilots notably improved, indicating better function probably through reduced airway obstruction, enhanced efficiency, and increased ventilation (David and Edwards, 2022; Pellegrino et al., 2005). In the control group, no significant change appeared; higher post-intervention SD suggested increased variability influenced by factors beyond intervention.

Significant FEV6% differences appeared in the intervention and control groups post intervention, with yoga group’s difference surpassing control’s; FEV6% effect size was large for both groups. In the control group, higher values and SD hint at external factors, yielding greater variation, with unrelated elements possibly impacting results, heightening participant variability. Yoga group’s FEV6% decrease with low SD implies consistent response to intervention, possibly due to controlled exhalation affecting air distribution over exhalation phases. Between groups, yoga’s significant p-value indicates substantial intervention-induced FEV6% impact, signaling significant respiratory function change (David and Edwards, 2022; Pellegrino et al., 2005).

FEV1% and FEV6% changes could arise from adjusted breathing patterns and varied exhalation, influenced by individual factors (Pellegrino et al., 2005; Wang et al., 2018). Effects of training might diverge among individuals. Techniques emphasizing calm, slow breathing could boost lung capacity through fuller expansion and efficient gas exchange. While the capacity increase lacked statistical significance, it could aid FEV1% improvement, facilitating air exhalation within the first second. Conversely, yoga group’s FEV6% reduction might connect to altered breathing patterns, potentially affecting initial six-second exhaled air, linked to strengthened respiratory muscles from yoga’s pranayama (Abel et al., 2013; Dinesh et al., 2015; Dubey et al., 2022; Mandanmohan et al., 2003; Sheetal et al., 2012; Thangavel et al., 2014) and control group’s military training (MacIntyre et al., 1981).

In the study’s context, the Borg CR10 scale provides insights into intervention group participants’ perceived exertion of the yoga class (Scherr et al., 2013). Comparing pre- and post-12-week perceptions with a paired t-test revealed a significant negative shift, indicating reduced exertion post-yoga. The low p-value underscores this change’s statistical significance. Cohen’s d effect size suggests a moderate effect favoring decreased effort after yoga. Participants experienced less strain during physical activity, even with increased challenges (advanced ásanas and longer holds). These findings, along with lung function results, suggest yoga’s positive impact on pilot’s physical capabilities: lower perceived exertion, combined with improved lung function and control, may imply enhanced adaptability and efficiency during physical tasks, including maneuvers like the anti-g maneuver by military pilots. Specific Pranayama types could stretch alveoli’s stretch receptors more, requiring strong control that delays fatigue onset (Prakash et al., 2007; Seltmann et al., 2020), possibly explaining decreased Borg values in the yoga group after 12 weeks.

### General Health Subjective Assessment (SF-36 V1 questionnaire)

Pre-intervention mean scores for overall health, as measured by the SF-36 questionnaire total score, were comparable between the control group and the yoga group. After the intervention, while the intervention group demonstrated a slight improvement, a significant decrease in overall health SF-36 scores was observed in the control group. The paired Cohen’s d between the control group before and after the intervention indicates a moderate effect size.

Conversely, the paired Cohen’s d between the intervention group before and after yoga indicates a small effect size. These effect sizes suggest a notable difference in the change of overall health SF-36 scores between the two groups, with the control group experiencing a larger decline in score when compared to the yoga group.

The modest improvement in overall health SF-36 scores among the intervention group suggests that the integration of yoga practices may mitigate the adverse health challenges associated with military training. The observed decline in overall health SF-36 scores among the control group underscores the potential challenges and stressors inherent in military training.

### Limitations

The study recognizes the limitations of the small sample size’s potential impact on result robustness, yet it’s essential to consider that the sample represents 100% of the specific population being studied. Working with focused populations often entails smaller sample sizes. Furthermore, due to COVID-related interruptions in Airforce class scheduling, the population size nearly doubled during data collection, presenting a distinct situation from its usual size.

### The Practical Applications of this Study Include

By improving muscle strength, breathing control, and with a reduced sense of effort, pilots can maintain better control and alertness during flights and even during physically challenging activities, demanding maneuvers, or flying high-G aircrafts. Additionally, biofeedback allows pilots to monitor and fine-tune these aspects right after their missions, further boosting their performance self-awareness, making their mission debrief process more accurate.

For F16 pilots transitioning to active flying, the Anti-G Straining Maneuver (AGSM) employs a breathing pattern to counter high gravitational forces’ effects. AGSM prevents G-induced loss of consciousness (G-LOC) by raising blood pressure and avoiding blood pooling (Dinesh et al., 2015). Yoga’s positive impact on lung capacity and control, as seen in the study, holds practical relevance for AGSM:

- Enhanced lung function, indicated by increased FEV1% and a large effect size, could aid anti-g maneuver execution.
- Improved airflow dynamics during early forced expiration supports generating necessary pressure against heightened G-forces. Yoga’s altered FEV6% exhalation patterns may bolster anti-g straining techniques.
- Incorporating yoga into military pilot training, particularly high-G operations, could enhance lung capacity and respiratory control. This could improve anti-g maneuver performance, mitigating G-LOC risk for mission safety and success.

For the Masters in Military Aeronautics Course - The increased physical capabilities can lead to better handling of aircrafts, allowing for smoother maneuvers and precise execution of military exercises. The improved control and alertness can lead to more focused and insightful debriefs, fostering better learning outcomes and performance refinement. Ultimately, the integration of these physical enhancements into the course can lead to an overall improvement in training quality, better preparing military pilots for the challenges of aviation operations.

For the Portuguese Airforce in general - all these aspects help in enhanced flight safety, reduction unnecessary maneuvers, conservation of fuel, and ultimately saving of tax money.

This investigation has some interesting practical applications that also can go onto the broader setting of sports exercise, an interesting approach for future investigation:

- Athletes can benefit from enhanced muscle strength and optimized breathing techniques. These enhancements enable athletes to control, focus, and perform across diverse sports, including physically demanding activities, crucial game moments, and endurance-based events.
- Integrating these findings into coaching and training programs can bring transformative results. Athletes with better muscle strength and refined breathing techniques can exhibit improved performance, better execution of techniques, more strategic decision-making under pressure, and more insightful post-training analyses. Enhanced control, alertness, and refined conversations with sports coaches can contribute to optimized learning and performance refinement.
- For the broader sports and fitness industry these findings mean that it’s possible to elevate safety standards during sports activities and training, leading to more effective and efficient workouts, reduced risk of injuries, and better overall athlete well-being.

## Conclusions

This study highlights the integration of yoga as an adjunct intervention to enhance strength, underscoring its holistic approach to physical wellness. The practice of yoga seems to also influence forced expiration volume, possibly due to increased respiratory muscle strength and endurance from pranayama training. These findings emphasize yoga’s advantages in optimizing physical performance and well-being in military aviation settings.

## Data Availability

Due to privacy concerns and regulations regarding sensitive information related to military personnel, the data collected for this study, including pilot-specific data, will not be made publicly available. We are committed to upholding the privacy and confidentiality of the individuals involved in our research.
